# The Effects of New Zealand Grown Ginseng Fractions on Cytokine Production from Human Monocytic THP-1 Cells

**DOI:** 10.3390/molecules26041158

**Published:** 2021-02-22

**Authors:** Wei Chen, Prabhu Balan, David G. Popovich

**Affiliations:** 1 School of Food and Advanced Technology, Massey University, Palmerston North 4442, New Zealand; W.Chen2@massey.ac.nz; 2 Riddet Institute, Massey University, Palmerston North 4442, New Zealand; balanprabhu@gmail.com; 3 Alpha-Massey Natural Nutraceutical Research Centre, Massey University, Palmerston North 4442, New Zealand

**Keywords:** *Panax ginseng*, NZ-grown ginseng, saponins, less-polar ginsenosides, pro-inflammatory, anti-inflammatory

## Abstract

Pro-inflammatory cytokines and anti-inflammatory cytokines are important mediators that regulate the inflammatory response in inflammation-related diseases. The aim of this study is to evaluate different New Zealand (NZ)-grown ginseng fractions on the productions of pro-inflammatory and anti-inflammatory cytokines in human monocytic THP-1 cells. Four NZ-grown ginseng fractions, including total ginseng extract (TGE), non-ginsenoside fraction extract (NGE), high-polar ginsenoside fraction extract (HPG), and less-polar ginsenoside fraction extract (LPG), were prepared and the ginsenoside compositions of extracts were analyzed by HPLC using 19 ginsenoside reference standards. The THP-1 cells were pre-treated with different concentrations of TGE, NGE, HPG, and LPG, and were then stimulated with lipopolysaccharide (LPS). The levels of pro-inflammatory cytokines, including tumor necrosis factor-alpha (TNF-α), interleukin-1 beta (IL-1β), interleukin-6 (IL-6), interleukin-8 (IL-8), and anti-inflammatory cytokines, such as interleukin-10 (IL-10), and transforming growth factor beta-1 (TGF-β1), were determined by enzyme-linked immunosorbent assay (ELISA). TGE at 400 µg/mL significantly inhibited LPS-induced TNF-α and IL-6 productions. NGE did not show any effects on inflammatory secretion except inhibited IL-6 production at a high dose. Furthermore, LPG displayed a stronger effect than HPG on inhibiting pro-inflammatory cytokine (TNF-α, IL-1β, and IL-6) productions. Particularly, 100 µg/mL LPG not only significantly inhibited the production of pro-inflammatory cytokines TNF-α, IL-1β, and IL-6, but also remarkably enhanced the production of anti-inflammatory cytokine IL-10. NZ-grown ginseng exhibited anti-inflammatory effects *in vitro*, which is mainly attributed to ginsenoside fractions (particularly less-polar ginsenosides) rather than non-saponin fractions.

## 1. Introduction

Inflammation is a protective response of the human body to harmful stimuli, such as microbial infections and chemical toxins [1]. It is a complex biological reaction involving coordinating signaling pathways and producing inflammatory cytokines and chemokines [2]. Cytokines are small extracellular proteins that act as mediators in the immune response. They regulate the inflammatory process through highly complex pathways [3]. It has already been reported that chronic low-grade inflammation and invigoration of the innate immune system are closely related to the pathogenesis of chronic diseases, such as diabetes [4], ageing [5], cancer [6], and cardiovascular disease [7]. Pro-inflammatory cytokines including interleukin-1 beta (IL-1β), interleukin-6 (IL-6), interleukin-8 (IL-8), and tumor necrosis factor-alpha (TNF-α), and anti-inflammatory cytokines such as interleukin-10 (IL-10), and transforming growth factor beta-1 (TGF-β1), have been reported to be involved in the events of inflammation-related diseases [8,9,10].

Ginseng is a popular Chinese herb. Traditionally, it is used for enhancing immunity, restoring homeostasis, treating illness, and helping longevity [11]. Ginsenosides, a type of triterpene saponins, are thought to be the unique and mainly active compositions of ginseng. Nearly two hundred ginsenosides have been identified from ginseng plants and products [12]. Recently, ginsenosides have been reported to show various beneficial effects, including anti-ageing [13], anti-fatigue [14], anti-cancer [15], anti-diabetes [16], and anti-inflammatory effects [17]. In New Zealand (NZ), *Panax ginseng* has been grown under the open-wild pine forest with the volcanic pumice soil and high-intensity UV rays. From our previous studies, NZ-grown ginseng contains abundant ginsenosides; the average content of total ginsenosides in NZ is much higher than that in other countries [18]. However, there are no publications about any bioactive effects of NZ-grown ginseng. Whether NZ-grown ginseng affects the production of pro-inflammatory or anti-inflammatory cytokines is not known.

The human monocytic THP cell line is derived from the peripheral blood of a 1-year-old male patient with acute monocytic leukemia [19]. This cell line has been widely used as a model to study immune response and functions. Usually, lipopolysaccharide (LPS) is used as an initiator to activate immune cells in vitro and in vivo studies [20]. In this study, four NZ-grown ginseng fractions, including total ginseng extract (TGE), non-ginsenoside fraction extract (NGE), high-polar ginsenoside fraction extract (HPG), and less-polar ginsenoside fraction extract (LPG), were prepared and analyzed by HPLC. LPS-induced THP-1 cells were used to evaluate the effects of TGE, NGE, HPG, and LPG on pro-inflammatory and anti-inflammatory cytokine productions. The results show that the ginsenoside fraction, rather than the non-ginsenoside fraction, exhibited the anti-inflammatory activity. Furthermore, LPG displayed a stronger effect than HPG on inhibiting pro-inflammatory cytokine (TNF-α, IL-1β, and IL-6) production. Particularly, 100 µg/mL LPG not only significantly inhibited the production of pro-inflammatory cytokines TNF-α, IL-1β, and IL-6, but also remarkably enhanced the production of anti-inflammatory cytokine IL-10. This study provides a preliminary explanation that LPG from NZ-grown ginseng can regulate the production of pro- and anti-inflammatory cytokines, suggesting LPG may have some potential to treat inflammation-related diseases. This needs to be further investigated in the future.

## 2. Results

### 2.1. Ginsenoside Profiles of Ginseng Extract Fractions

**Figure 1:**
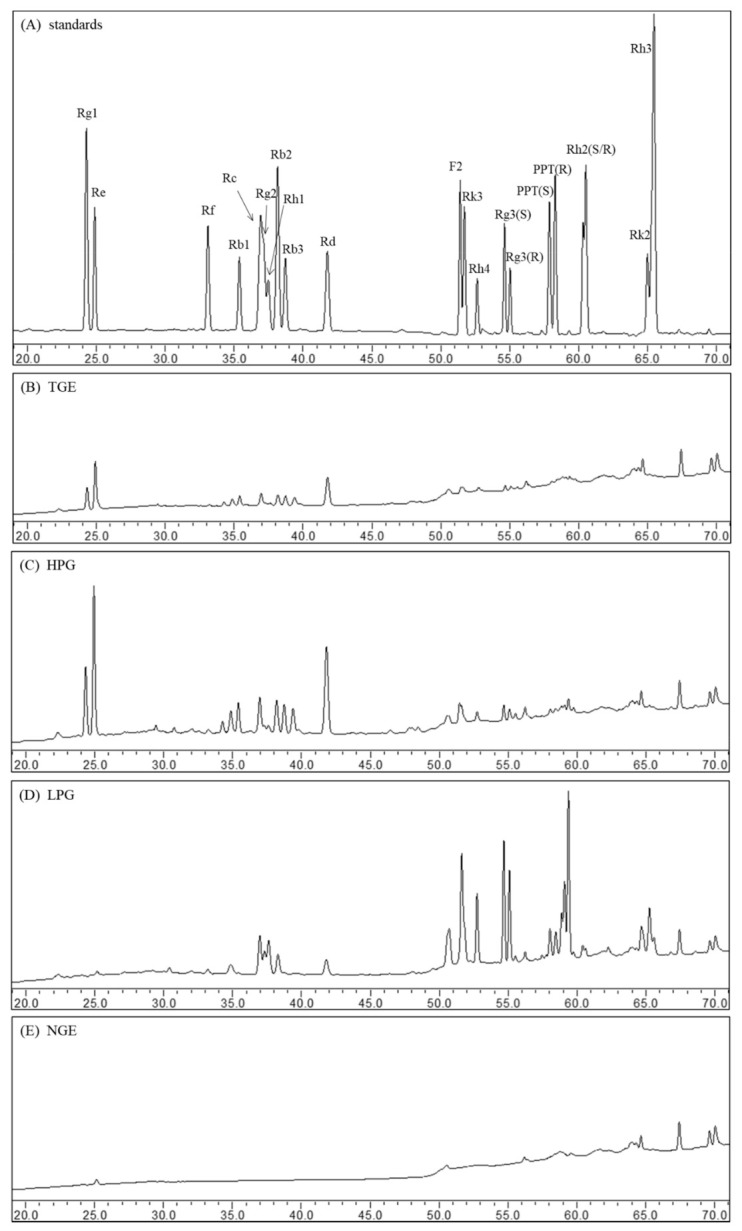
The HPLC chromatograms of ginsenoside standards (**A**), TGE (**B**), HPG (**C**), LPG (**D**), and NGE (**E**).

The ginsenosides were separated within 70 min by the RP-C18 column using HPLC. The peaks of individual ginsenosides appeared between 20 min and 70 min in HPLC chromatograms. The ginsenoside profiles of ginsenoside standards and different fraction extracts are shown in Figure 1. The main compositions of TGE are high-polar ginsenosides, such as ginsenosides Rg1, Re, Rc, and Rd (Figure 1B). Compared to the profile of TGE, the ginsenoside composition of HPG did not change after *n*-butanol liquid-liquid extraction; they displayed similar ginsenoside peaks, but the height and area of peaks increased after *n*-butanol liquid-liquid extraction (Figure 1C). In the water fraction extract, no ginsenoside was detected (Figure 1E). This suggests that the ginsenoside composition and non-ginsenoside composition were effectively separated through *n*-butanol liquid-liquid extraction. From the HPLC profile of LPG, we can see that the peaks of high-polar ginsenosides (including Rg1, Re, Rb1) disappeared. Notably, some peaks of less-polar ginsenosides (such as Rg3, Rk3, Rh4) emerged during the 50–70 min (Figure 1D). This means high-polar ginsenosides were converted into less-polar ginsenosides through high-temperature treatment.

### 2.2. Quantification of Ginsenosides in the Ginseng Fraction Extracts

The concentrations of ginsenosides in different ginseng fraction extracts were quantified through HPLC. The contents of 19 individual ginsenosides and the total ginsenosides are listed in Table 1. TGE and HPG contain the same compounds, and ginsenosides Re and Rd were the two major compounds in both extracts, while HPG had a much higher concentration of ginsenosides compared to TGE. The total content of ginsenosides in HPG (360.00 ± 3.17 mg/g) increased 2.4 times after removing non-ginsenoside components from TGE (105.39 ± 11.96 mg/g) through liquid-liquid extraction. In the LPG extract, the common high-polar ginsenosides such as Rb1, Rg1, Re, and Rc were not detected. The majority of the components were less-polar ginsenosides; its contents reached 424.85 ± 2.60 mg/g. Ginsenoside Rg3 (20S/R Rg3), which is regarded as the unique active compound in ginseng products [21,22], was enriched and accounted for 27.6% in the LPG extract.

### 2.3. The Effect of Different Ginseng Fraction Extracts on Cell Viability

**Figure 2:**
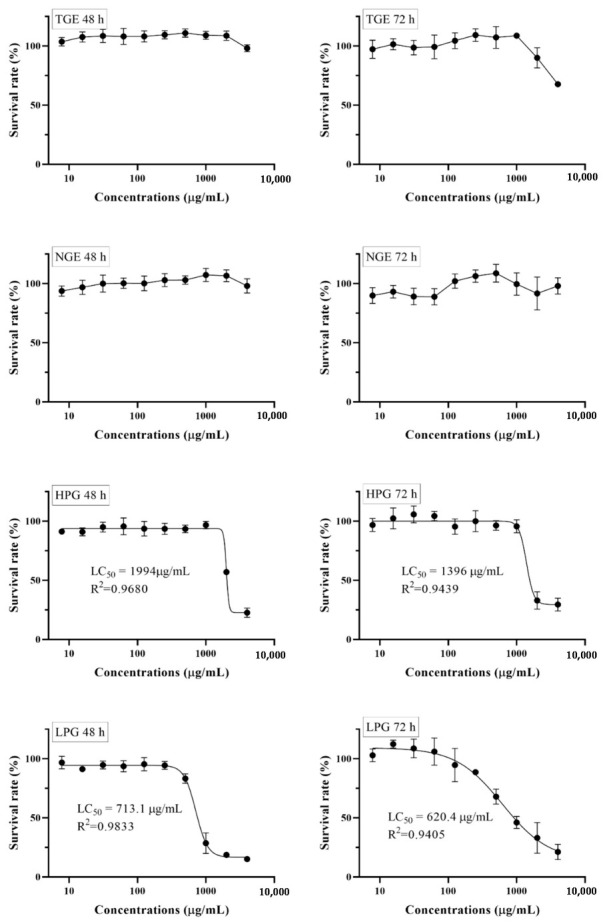
MTT dose-response relationship of ginseng fraction extracts after 48 and 72 h incubation with THP-1 cells assessed by an MTT viability assay. Values are expressed as a percentage of untreated control cells (mean ± SD).

To evaluate the cytotoxic effect of fractioned ginseng extracts on THP-1 cells, an MTT assay was carried out; dose-response curves are shown in Figure 2. The horizontal axis is concentrations of tested extract, and the vertical axis is the survival rate of cells treated with different concentrations of tested extract for 48 h or 72 h. There was no obvious toxic effect of TGE and NGE on cell growth below 1000 µg/mL. At low concentrations, there was no obvious effect on cell growth, but when concentrations increased to some extent, HPG and LPG showed a dose-dependent response and decreased cell growth as concentrations increased. A best-fit curve (four parameter curve) was drawn. The LC_50_ (lethal concentration 50%) values were determined by plotting cell viabilities against concentrations. The LC_50_ of HPG for 48 h and 72 h is 1994 µg/mL (R^2^ = 0.9680) and 1396 µg/mL (R^2^ = 0.9439), respectively, while the LC_50_ of LPG for 48 h and 72 h is 713.1 µg/mL (R^2^ = 0.9833) and 620.4 µg/mL (R^2^ = 0.9405), respectively. The results show that the toxicity to THP-1 cells increased when high-polar ginsenosides converted to less-polar ginsenosides. Cells seemed more sensitive to the less-polar ginsenosides. Additionally, Figure 2 shows that the concentrations below 250 µg/mL of LPG and HPG, and the dose under 1000 µg/mL of TGE and NGE were not toxic to THP-1 cells treated for 48 h. Thus, advisable concentrations (1, 10, and 100 µg/mL for HPG and LPG; 10, and 100 µg/mL for NGE; 40, and 400 µg/mL for TGE) were used in the further assay.

### 2.4. The Effect of Different Ginseng Fraction Extracts on Cell Viability

**Figure 3:**
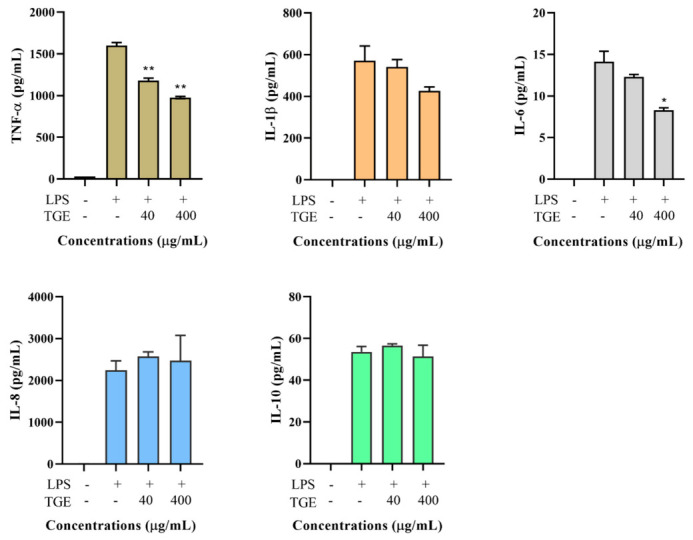
Effect of TGE on LPS-induced TNF-α, IL-1β, IL-6, IL-8, and IL-10 productions in the THP-1 cell culture medium. Data are shown as the mean ± SD of three repetitions. * *p* < 0.05 and ** *p* < 0.01 for comparison of LPS stimulation with and without ginseng extract pre-treatment.

Based on the concentration of ginsenosides in the TGE and MTT assay, 40 and 400 µg/mL TGE were used to evaluate the effect of total ginseng extract on cytokine production. TGE significantly inhibited LPS-stimulated TNF-α production from 1600 ± 38 pg/mL to 1180 ± 31 pg/mL at a concentration of 40 µg/mL, and to 978 ± 14 pg/mL at a concentration of 400 µg/mL, respectively. There was an inhibited trend for IL-1β and IL-6 production in TGE treatment groups compared to control groups, but no statistical significance except for IL-6 secretion treated with 400 µg/mL of TGE. There was no significant difference in other cytokine productions whether it was treated with TGE or not (Figure 3).

### 2.5. Effects of Non-Ginsenoside Fraction Extract (NGE) on Cytokine Production

**Figure 4:**
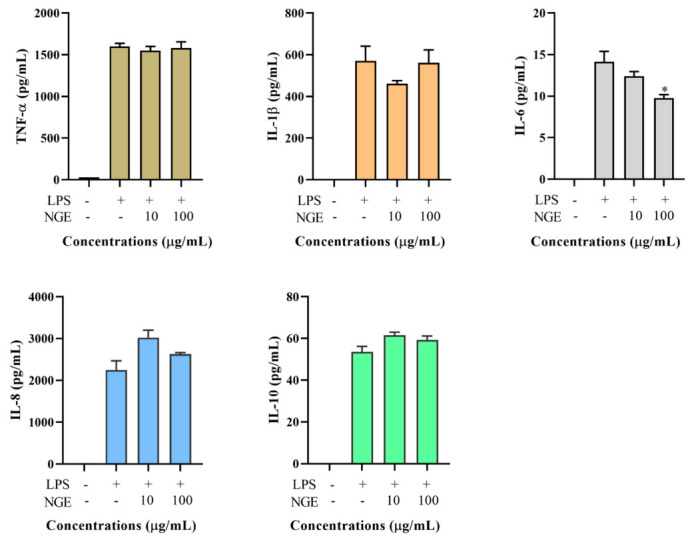
Effect of TGE on LPS-induced TNF-α, IL-1β, IL-6, IL-8, and IL-10 productions in the THP-1 cell culture medium. Data are shown as the mean ± SD of three repetitions. * *p* < 0.05 for comparison of LPS stimulation with and without ginseng extract pre-treatment.

Total ginseng extract contains ginsenoside and non-ginsenoside fractions. It showed that the non-ginsenoside fraction accounted for about three-fourths of the total ginseng extract through the separated process (see method section). However, the non-ginsenoside fraction may not be the active ingredient of ginseng, although its concentration is high. As shown in Figure 4, NGE, the major component of ginseng extract, had no significant effect on the production of various cytokines, except for IL-6. In particular, the total ginseng extract could decrease TNF-α production by 26.3–38.9% (*p* < 0.05), but NGE, fractioned from the total ginseng extract, did not show any obvious effect on TNF-α production.

### 2.6. Effects of Ginsenoside Fraction Extracts on Cytokine Production

**Figure 5:**
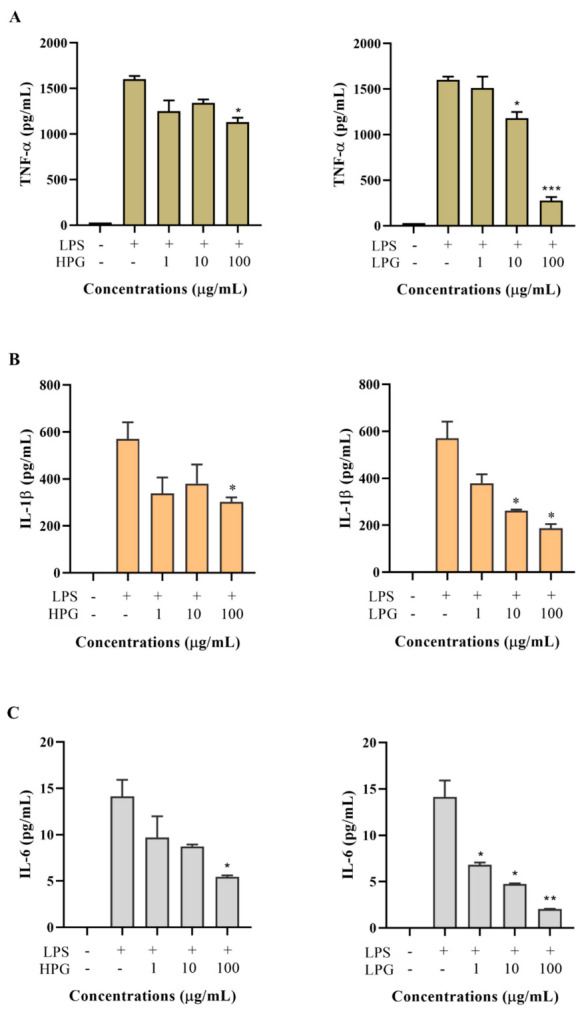

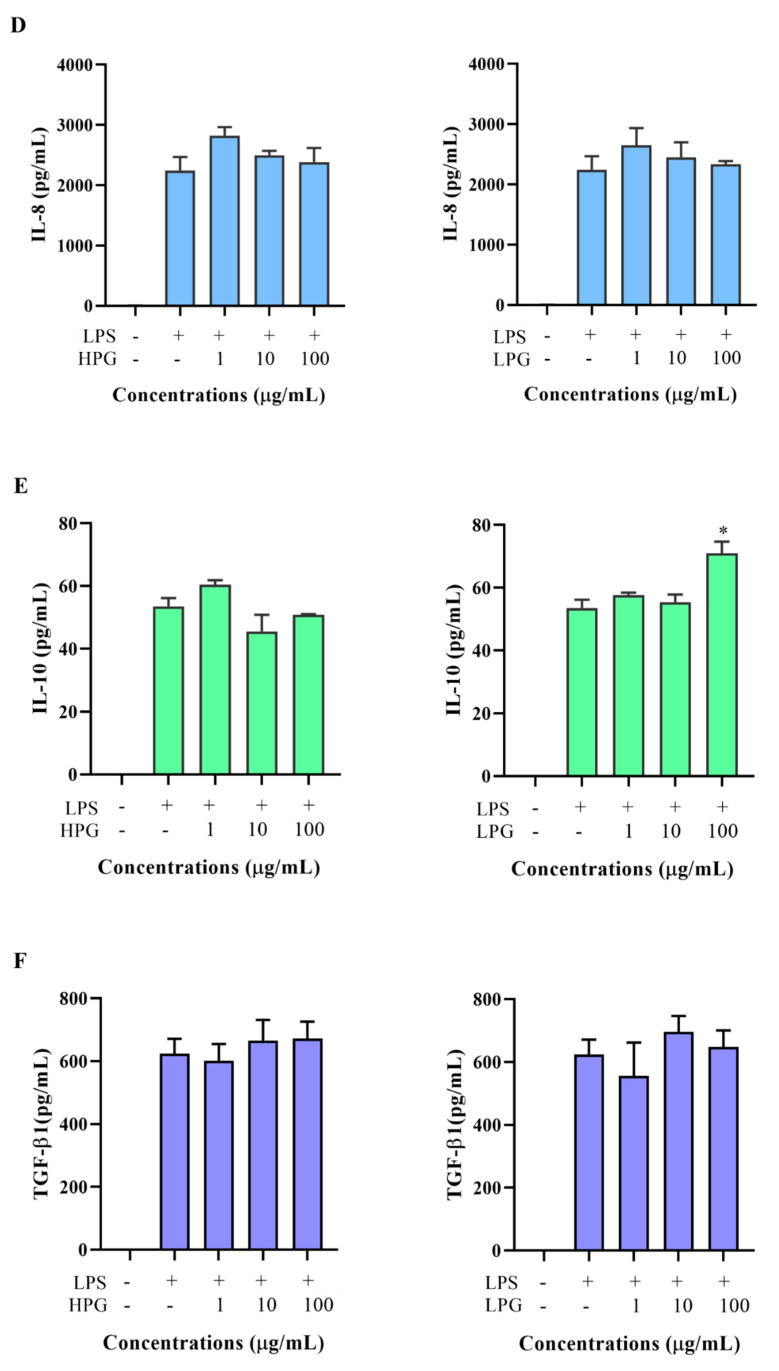
Effect of HPG and LPG on LPS-induced TNF-α (**A**), IL-1β (**B**), IL-6 (**C**), IL-8 (**D**), IL-10 (**E**), and TGF-β1 (**F**) production in the THP-1 cell culture medium. Data are shown as the mean ± SD of three repetitions. * *p* < 0.05, ** *p* < 0.01, and *** *p* < 0.001 for comparison of LPS stimulation with and without ginseng extract pre-treatment.

Ginsenosides are commonly regarded as the active ingredient of ginseng. High-polar ginsenoside fraction extracts (HPG) and less-polar ginsenoside fraction extracts (LPG) were separated from a total ginseng extract. To evaluate the effect of HPG and LPG on cytokine production, THP-1 cells were treated with various concentrations of HPG and LPG for 2 h, followed by LPS (200 ng/mL) incubation. 24 h after LPS treatment, cytokines including TNF-α, IL-1β, IL-6, IL-8, IL-10, and TGF-β1 in the culture medium were determined. Results are shown in Figure 5. Five cytokines (TNF-α, IL-1β, IL-6, IL-8, and IL-10) were almost undetectable in the culture medium without PLS treatment, but their levels increased dramatically after LPS stimulation. A high dose (100 µg/mL) rather than a low dose (10 or 1 µg/mL) of HPG significantly inhibited LPS-induced TNF-α, IL-1β, and IL-6 productions. LPG showed a dose-dependent inhibitory effect on LPS-induced TNF-α, IL-1β, and IL-6 productions. 100 µg/mL LPG significantly decreased LPS-stimulated TNF-α from 1600 ± 38 pg/mL to 275 ± 41 pg/mL, IL-1β from 571 ± 50 pg/mL to 188 ± 12 pg/mL, and IL-6 from 14.1 ± 1.3 pg/mL to 2.1 ± 0.1 pg/mL, respectively (Figure 5A–C). Both HPG and LPG did not show significant effects on LPS-induced IL-8 and TGF-β1 productions at different concentrations from 1 µg/mL to 100 µg/mL (Figure 5D,F). Interestingly, LPG at 100 µg/mL significantly enhanced LPS-induced IL-10 production, while HPG at different doses did not exhibit an obvious effect on LPS-stimulated IL-10 production (Figure 5E).

## 3. Discussion

Pro-inflammatory or anti-inflammatory cytokines can be produced by leukocytes or endothelial cells based on their activities in regulating inflammation [23]. TNF-α and IL-1β, two pro-inflammatory cytokines, have been identified as playing a central role in acute and chronic inflammation [24,25]. Anti-inflammatory cytokines, such as IL-10 and TGF-β, negatively regulate or inhibit over-activated inflammation [26]. Therefore, either suppressing the overproduction of pro-inflammatory cytokines or enhancing the production of anti-inflammatory cytokines is an effective strategy to reduce inflammation and its symptoms, and this method has been reported to successfully treat certain inflammatory diseases, such as diabetes, rheumatoid arthritis, and cancer [27,28]. The total saponin extracts from *Panax ginseng* have shown anti-inflammatory effects through reducing productions of nitric oxide (NO) and LPS-induced TNF-α and IL-1β protein expression [28], which is in line with the present study that total extracts from NZ-grown ginseng suppressed LPS-induced TNF-α, IL-1β, and IL-6 production. Furthermore, in this work, it was demonstrated that ginsenoside fractions rather than non-ginsenoside fractions are the main component of ginseng to exert anti-inflammatory activity. This result can be supported by previous publications. It has been reported that the ginsenosides Rg1 [29], Re, Rb1, Rd [30], Rc [31], Rh3 [32], and Rg3 [33] are able to inhibit the production of pro-inflammatory cytokines, including TNF-α and IL-1β, in in vitro and *in vivo*. From the HPLC analysis, the main ingredients of ginsenoside fractions from NZ-grown ginseng are ginsenoside Rb1, Rg1, Re, Rg3. This suggests that ginsenosides are the anti-inflammatory components of NZ-grown ginseng.

From the present study, LPG exhibited more inhibition of pro-inflammatory cytokine secretion compared with HPG at the same dose, suggesting that the anti-inflammatory activity of HPG is enhanced after transforming into LPG by a heating process. Similar results can be found in the literature. It has been reported that black ginseng extract [34] and fermented red ginseng extract [35] possess more potent anti-inflammatory and anti-nociceptive activities than red ginseng extract. Interestingly, black ginseng and fermented red ginseng mainly contain LPG, while the majority component of red ginseng is HPG [36]. This may be due to the smaller molecule structure of LPG compared to HPG, which makes it easier to pass through the cell membrane to exhibit relatively higher biological activities [37]. In the present study, LPG inhibited the production of LPS-induced pro-inflammatory cytokine of TNF-α, IL-1β, and IL-6, on the other hand, enhanced LPS-induced anti-inflammatory cytokine of IL-10 secretion in THP-1 cells. This indicates that LPG possesses a more effective anti-inflammatory effect than HPG, which shows an inhibitory effect on pro-inflammatory cytokine production, but HPG has no obvious effect on anti-inflammatory cytokine production. Together with previous studies, the results obtained with this work might indicate that ginseng extract from NZ-grown ginseng also shows an anti-inflammatory effect and that processed ginseng extract (LPG) might have potential application in regulating inflammatory diseases.

Additionally, the amount of ginseng extract that does not contain ginsenosides (named non-ginsenosides fraction extract, NGE) was obtained from total ginseng extracts in this study. Although ginsenosides are commonly considered a unique component of ginseng and attract much more attention from researchers, non-saponins account for most of the chemical components of ginseng [38]. Non-saponins of ginseng can be classified into three categories: saccharides including monosaccharide, oligosaccharides, polysaccharides, fiber, and pectin; Nitrogen-containing compounds such as amino acids, peptides, protein, alkaloids, and nucleic acids; and fat-soluble components including polyacetylenes, fatty acid, lipids, essential oil, organic oil, phenolic compounds, terpenoid, and phytosterols [38]. Considering the extraction method of this study, the major ingredient of NGE is saccharides, most likely ginseng polysaccharides. Kang et al. found that non-saponin fractions (NSF) from red ginseng significantly decreased pro-inflammatory cytokine, including TNF-α and IL-6, and monocyte chemoattractant protein 1 (MCP-1) [39]. Moreover, NSF successfully inhibited inflammatory responses by reducing the productions of NO, inducible nitric oxide synthase (iNOS), and cyclooxygenase-2 (COX-2). For example, 2000 µg/mL NSF reduced the levels of NO, iNOS, and COX-2 by 33%, 83%, and 64%, respectively [39]. Another study evaluated the anti-diabetic effect of KGC05P0, which is a non-saponin fraction of Korean red ginseng. They found that KGC05P0 could regulate diabetes-related indicators including HbA1c, insulin, glucose tolerance, and fasting glucose level, via downregulating the PI3K/AKT pathway, inhibiting gluconeogenesis [40]. In addition, 400 mg/kg KGC05P0 significantly reduced TNF-α and IL-1β levels in blood from diabetic mice [40]. Besides the study on non-saponin fractions, one study focused on red ginseng acidic polysaccharide (RGAP) and found that RGAP treatment could raise the levels of IL-1, IL-6, and NO by macrophages, whereas TNF-α production did not differ between RGAP treatment or no RGAP treatment [41], which is similar to the results in this study. Only 100 µg/mL NGE inhibited LPS-induced IL-6 production; NGE did not show effects on other cytokine productions in the present study. This is likely because the concentration (10–100 µg/mL) in this study was relatively low compared to the 400 mg/kg [40] or 2000 µg/mL [39] used in other studies. A recent study found that non-saponin fractions with rich polysaccharides of ginseng showed beneficial effects against aging and Alzheimer’s disease. The dose of non-saponin fractions used in that study were 500 and 1000 µg/mL. These suggest that a higher concentration of NGE should be used to explore bioactivities and functions of non-saponin fraction extracts from NZ-grown ginseng in the future.

## 4. Materials and Methods

### 4.1. Chemicals

Twenty-one reference standards of ginsenosides, including Rg1, Re, Rf, Rb1, Rc, Rg2, Rh1, Rb2, Rb3, Rd, F2, Rk3, Rh4, 20(S)-Rg3, 20(R)-Rg3, 20(S)-PPT, 20(R)-PPT, 20(S)-Rh2, 20(R)-Rh2, Rk2, and Rh3, were purchased from Star Ocean Ginseng Ltd. (Suzhou, Jiangsu, China). The purities of all reference standards were no less than 98.0%. RPMI 1640 medium, fetal bovine serum (FBS), penicillin, and streptomycin were purchased from Gibco Life Technologies (Grand Island, NY, USA). The LPS (*Escherichia coli* 0127: B8), dimethyl sulfoxide (DMSO), 2-mercaptoethanol, were purchased from Sigma-Aldrich (St Louis, MO, USA). Enzyme-linked immunosorbent assay (ELISA) kits for human TNF-α, IL-1β, IL-6, IL-8, IL-10, and TGF-β1 were purchased from Thermo Fisher Scientific (Waltham, MA, USA). Acetonitrile (HPLC grade) and *n*-butanol were purchased from Merck Life Science (Auckland, New Zealand). Water (deionized) was obtained from a Milli-Q Ultra-pure water system (Millipore, Billerica, MA, USA).

### 4.2. Ginseng Materials

Ginseng extract powder (whole NZ-grown ginseng plants were collected in May 2019 from pine forest around Taupo, NZ. After air-dried, the samples were extracted through water and the extract was filtered, concentrated, and dried into powder) was provided by Kiwiseng Co. Ltd. (Rotorua, NZ) and marked as TGE (total ginseng extract).

### 4.3. Cells

The human monocytic THP-1 cell line was purchased from the American Type Culture Collection (ATCC, Manassas, VA, USA).

### 4.4. Ginseng Fractions Preparation

4.85 g TGE was dissolved in 40 mL water. Then ginsenosides were extracted by liquid-liquid extraction using *n*-butanol. The ginseng extract aqueous solution was extracted three times with 30 mL water-saturated *n*-butanol. The *n*-butanol layer fraction, which consists of high-polar ginsenosides (HPG), and the water layer fraction (non-ginsenoside extract, NGE) were evaporated at 60 °C using a rotary vacuum evaporator, respectively.

**Figure 6:**
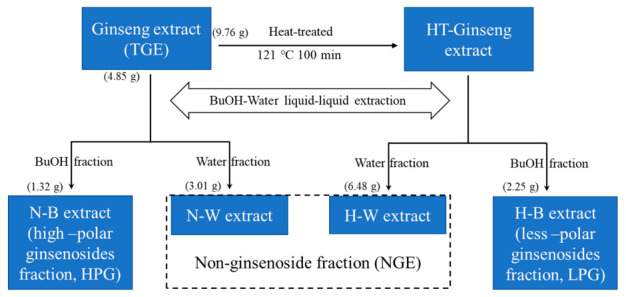
The flow chart of preparation of ginseng extract fractions.

TGE (9.76 g) was dissolved in 100 mL water and steamed in an autoclave (121 °C, 0.1 Mpa) for 100 min. Then the high temperature treated ginseng aqueous solution was extracted three times with 80 mL water-saturated *n*-butanol. The *n*-butanol layer fraction, containing less-polar ginsenosides (LPG), and the water layer fraction (NGE) were evaporated at 60 °C using a rotary vacuum evaporator, respectively. The concentrated extracts were freeze-dried into powder. Finally, 1.32 g HPG, 2.25 g LPG, and 9.49 g NGE (3.01 g N-W extract and 6.48 g H-W extract) were obtained and stored at –20 °C before use. The flow chart of ginseng extract fraction preparation is shown in Figure 6.

### 4.5. HPLC Analysis

The different ginseng extracts (TGE, HPG, LPG, and NGE) and twenty-one ginsenoside reference standards were prepared about 1 mg/mL using deionized water or 70% methanol, and then filtered through a 0.22-mm syringe filter before HPLC injection. The HPLC analysis, including validated calibration curves, was conducted as a previous study [42]. The HPLC instrument used in this study was a Shimadzu Prominence LC-20A UFLC Stack HPLC system (Shimadzu, Kyoto, Japan) equipped with a DGU-20A3 degasser, LC-20AD pump, CTO column oven, SPD-20A detector, and SIL-20A autosampler. A Waters Symmetry Shield RP18 column (4.6 mm × 250 mm, 5 μm) was used. The mobile phase consisted of deionized water (A) and acetonitrile (B). The gradient elution program was as follows: 0–15 min, 20% B; 15–25 min, 20–31% B; 25–35 min, 31–35% B; 35–40 min, 35–36% B; 40–45 min, 36–39% B; 45–75 min, 39–95%; 75–85 min, 95–20% B, 85–90 min, 20% B. The column temperature was set at 25 °C and the flow rate was 1 mL/min. The sample injection volume was 20 μL and the wavelength of detection was set at 203 nm.

### 4.6. THP-1 Cell Culture and Treatments

The THP-1 cells were cultured in RPMI 1640 medium containing 10% (*v*/*v*) FBS, 1% (*v*/*v*) antibiotics (100 U/mL penicillin and 100 µg/mL streptomycin), and 0.05 mM 2-mercaptoethanol in a humidified incubator at 37 °C containing 5% CO_2_. Cells were subcultured every 3 days and maintained at a concentration between 1 × 10^5^ and 1 × 10^6^ cells/mL. THP-1 cells (5 × 10^5^/mL) were incubated in 24-well, flat bottomed plates for 24 h. The cells were treated with TGE (40, and 400 µg/mL), NGE (10, and 100 µg/mL), HPG (1, 10, and 100 µg/mL), LPG (1, 10, and 100 µg/mL) or vehicle alone for 2 h, and then stimulated with 0.2 μg/mL LPS. The cell-free supernatants were collected 24 h after LPS simulation [43].

### 4.7. Cell Viability

Cell viability was evaluated using the 3-(4,5-dimethylthiazol-2-yl)- 2,5-diphenyltetrazolium bromide (MTT) assay [44]. Briefly, serial dilutions of ginseng extracts (TGE, NGE, HPG, and LPG) were added to each well of a 96-microwell plate. THP-1 cells were seeded to each well to a final density of 5 × 10^5^ cells/mL. Control wells contained THP-1 cells and culture medium, but no tested extracts. Cells were incubated for 48, and 72 h before adding MTT (0.5 mg/mL) and incubated in the dark for 4 h. Formazan crystals were solubilized for 30 min with DMSO. Optical density measurements were recorded at 550 nm absorbance in a Multiskan FC Microplate Photometer (Thermo Scientific, Waltham, MA, USA) for the 96-microwell plate.

### 4.8. Enzyme-Linked Immunosorbent Assay (ELISA)

The concentrations of TNF-α, IL-1β, IL-6, IL-8, IL-10, and TGF-β1 in supernatants were determined by ELISA kits according to the manufacturer’s instructions. A Multiskan FC Microplate Photometer (Thermo Scientific, Waltham, MA, USA) was used to detect the optical density at 450 nm. The standard curves of TNF-α, IL-1β, IL-6, IL-8, IL-10, and TGF-β1 see Figure S1.

### 4.9. Statistical Analysis

Statistical analyses were performed using GraphPad Prism 8 for Windows (GraphPad Software, Version 8.01, La Jolla, CA, USA). All data are expressed as means ± SD (*n* = 3). An unpaired *t*-test was used to analyze the experimental data. Differences were considered statistically significant at a *p*-value < 0.05.

## 5. Conclusions

This is the first preliminary study on the activity of NZ-grown ginseng. This in vitro study suggests that ginsenoside fractions, rather than non-ginsenoside fractions, inhibit the productions of LPS-induced pro-inflammatory cytokines. This inhibitory effect is enhanced when HPG converts into LPG by heat-treatment. Furthermore, LPG can not only inhibit LPS-induced pro-inflammatory cytokine production but also improve LPS-induced anti-inflammatory cytokine secretion.

## Figures and Tables

**Table 1:** Ginsenoside content (mg/g) in the TGE, HPG, and LPG extracts.

Ginsenosides	Ginsenoside Content (mg/g) of Ginseng Extracts		
	TGE	HPG	LPG
Rg1	12.10 ± 1.5	44.99 ± 0.49	
Re	29.08 ± 2.20	97.26 ± 2.08	
Rf	1.08 ± 0.66	5.44 ± 0.66	
Rb1	7.46 ± 0.70	25.40 ± 1.93	
Rc	10.63 ± 0.62	37.44 ± 0.95	
Rg2			26.75 ± 1.17
Rh1			29.71 ± 0.95
Rb2	10.12 ± 1.38	35.85 ± 0.89	
Rb3	8.77 ± 1.33	30.00 ± 0.09	
Rd	26.15 ± 3.56	83.62 ± 10.25	18.07 ± 1.79
F2			74.31 ± 2.15
Rk3			32.01 ± 2.18
Rh4			53.03 ± 0.44
20(S)-Rg3			51.95 ± 0.10
20(R)-Rg3			65.46 ± 0.12
20(S)-PPT			15.64 ± 0.03
20(S)-Rh2			4.43 ± 0.39
Rk2			43.27 ± 0.25
Rh3			10.23 ± 0.21
Total	105.39 ± 11.96	360.00 ± 3.17	424.85 ± 2.60

## Data Availability

The data presented in this study are available on request from the corresponding authors.

## Supplementary Materials

The following are available online, Figure S1: The standard curves of TNF-α, IL-1β, IL-6, IL-8, IL-10, and TGF-β1.

## Sample Availability

Samples of the compounds are not available from the authors.
