# Supplementary material for: The Effects of New Zealand Grown Ginseng Fractions on Cytokine Production from Human Monocytic THP-1 Cells
Source: Molecules. 2021 Feb 22;26(4):1158. doi: 10.3390/molecules26041158 (PMC7926829; doi:10.3390/molecules26041158)
Supplement: Supplementary file 1 [file molecules-26-01158-s001.pdf]

Supplementary data

TNF-α standard curve

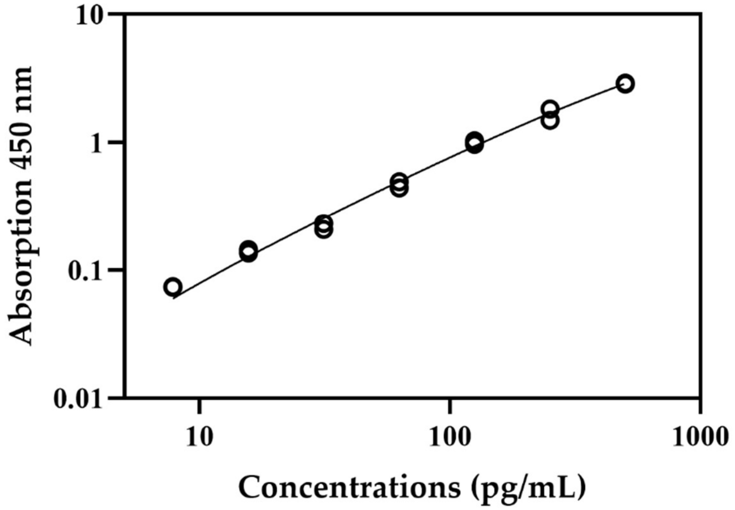

IL-1β standard curve

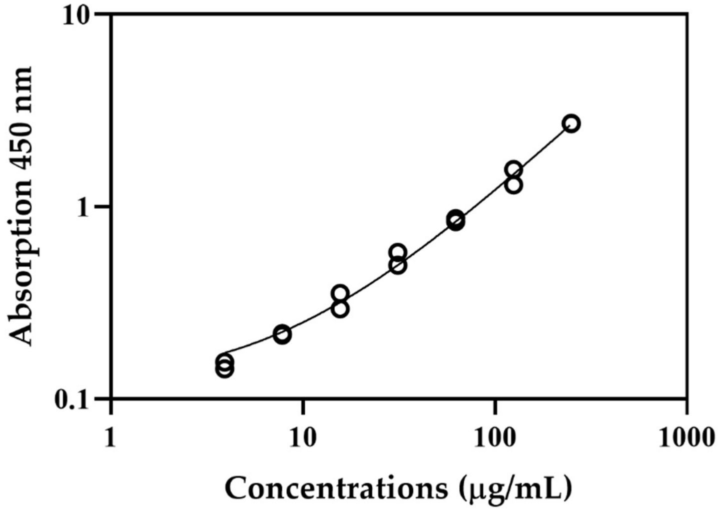

IL-6 standard curve

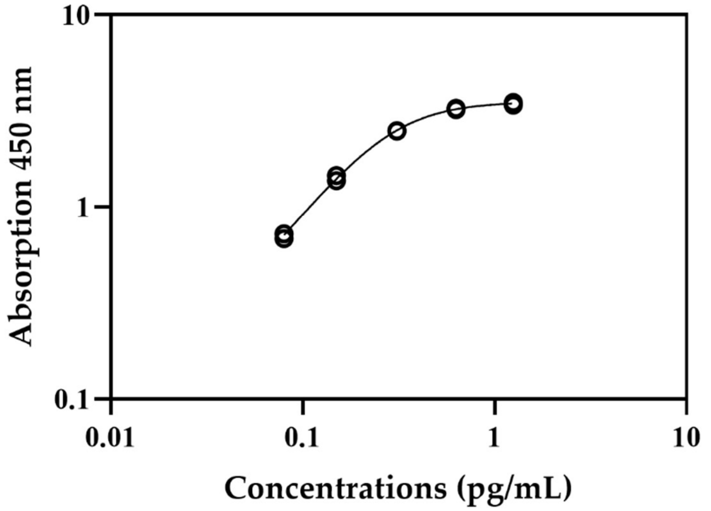

IL-8 standard curve

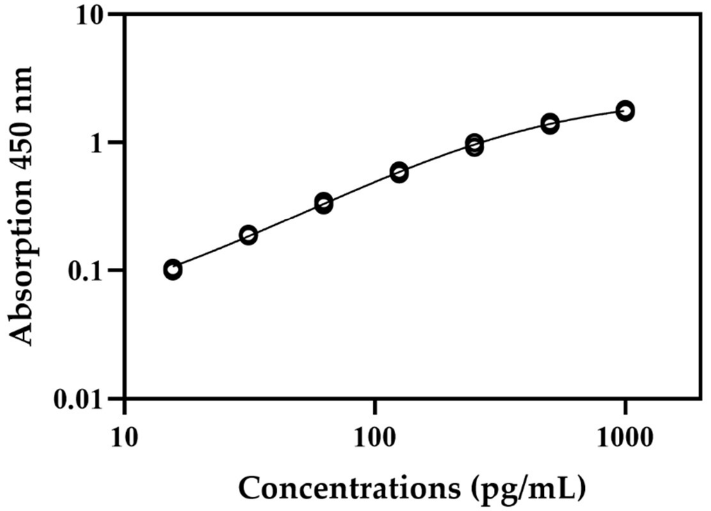

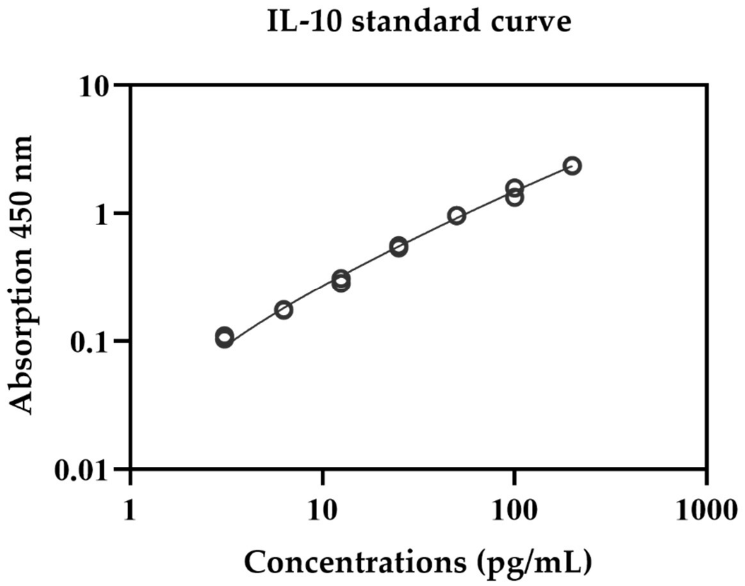

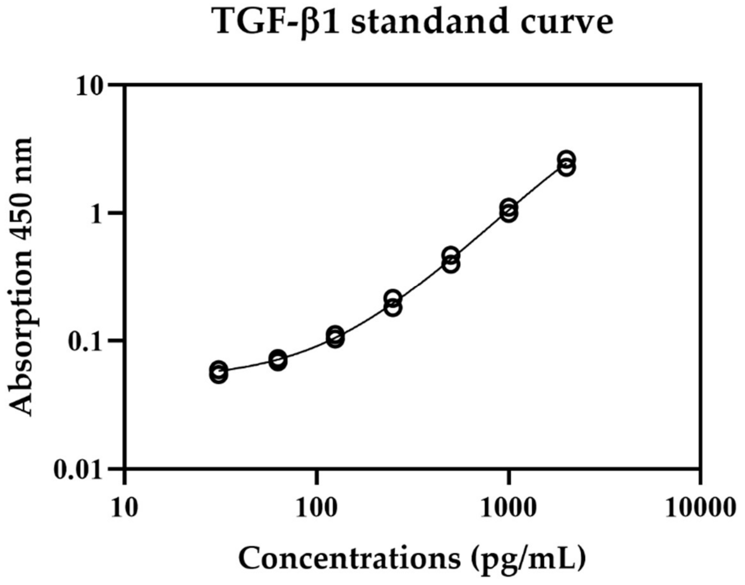

Figure S1: The standard curves of TNF-α, IL-1β, IL-6, IL-8, IL-10, and TGF-β1.
